# Downregulation of matrix metalloproteinase 9 by small interfering RNA inhibits the tumor growth of ovarian epithelial carcinoma *in vitro* and *in vivo*

**DOI:** 10.3892/mmr.2021.12391

**Published:** 2021-08-31

**Authors:** Fengjun Guo, Jingyan Tian, Manhua Cui, Meiru Fang, Lin Yang

Mol Med Rep 12: 753-759, 2015; DOI: 10.3892/mmr.2015.3425

Following the publication of this paper, the authors contacted the Editorial Office to request that the article be retracted on account of an inability to obtain consistent results after having repeated the experiments portrayed in Figs. 1B and 3B. Independently, it was drawn to the Editor's attention that certain of the western blotting data shown in these figures were strikingly similar to data appearing in different form in other articles by different authors.

Owing to the fact that these other articles were under consideration for publication at the same time as the above article was submitted for publication to *Molecular Medicine Reports*, the Editor has agreed to the authors’ request that this article should be retracted from the Journal. The Editor apologizes to the readership for any inconvenience caused.

